# Aloe‐Emodin Improves Mitophagy in Alzheimer's Disease via Activating the AMPK/PGC‐1α/SIRT3 Signaling Pathway

**DOI:** 10.1111/cns.70346

**Published:** 2025-03-24

**Authors:** Yulu Wang, Yunzhi Ge, Siyu Hua, Chenrui Shen, Biao Cai, Han Zhao

**Affiliations:** ^1^ College of Integrated Chinese and Western Medicine Anhui University of Chinese Medicine Hefei China; ^2^ Institute of Integrated Chinese and Western Medicine Anhui Academy of Chinese Medicine Hefei China; ^3^ Key Laboratory of Xin'an Medicine Anhui University of Chinese Medicine, Ministry of Education Hefei China; ^4^ Anhui Province Key Laboratory of Chinese Medicinal Formula Hefei China

**Keywords:** aloe‐emodin, Alzheimer's disease, AMPK/PGC‐1α/SIRT3, cognitive dysfunction, mitochondrial fission/fusion, mitochondrial function, mitophagy, Pink1/parkin

## Abstract

**Background:**

Impaired mitophagy results in the accumulation of defective mitochondria that are unable to be cleared effectively in Alzheimer's disease (AD). Aloe‐emodin (AE), a key component of the traditional Chinese medicine Rhubarb, exhibits neuroprotective effects against Alzheimer's disease, though the underlying mechanism remains unclear. Studying aloe‐emodin's role in enhancing mitophagy is vital for improving cognitive function and reducing neuronal damage in Alzheimer's disease.

**Methods:**

The APP/PS1 double transgenic mice were adopted as models for AD to assess the effects of aloe‐emodin upon cognitive function and its neuroprotective impact on hippocampal neurons. Additionally, we investigated the regulatory mechanisms of proteins within the aforementioned pathway, and the morphological characteristics of mitophagy‐related proteins. An AD hippocampal neuron model was developed using Aβ25‐35 to evaluate the mitochondrial function, the protein expression of such a pathway and the mitophagy. This approach aims to elucidate the effects and underlying mechanisms of aloe‐emodin in relation to AD.

**Results:**

AE activates mitophagy in neurons, improves cognitive dysfunction, reduces hippocampal damage, and alleviates AD symptoms in model mice. AE activates the expression of AMPK, PGC‐1α and SIRT3. Increased expression of SIRT3 in mitochondria promotes mitophagy and regulates the function of mitochondrial proteins. When mitochondrial autophagy is enhanced, the expression of Beclin1, LC3, P62, Parkin, and PINK1‐related proteins changes. Further in vitro experiments showed that AE can enhance mitochondrial function in Alzheimer's disease cell models. The mitochondrial membrane potential, GSH, ROS and Ca2+ levels gradually recover, alleviating the pathological manifestations of AD. Knocking down SIRT3 leads to increased mitochondrial damage and a reduction in mitophagy in HT22 cells.

**Conclusion:**

Experimental results show that AE can activate mitophagy through AMPK/PGC‐1α/SIRT3 pathway, alleviate cognitive dysfunction in AD, and reduce damage to hippocampal neurons.

## Graphical abstract

AE activates the AMPK/PGC‐1α/SIRT3 pathway. Increased expression of SIRT3 in mitochondria promotes mitophagy and regulates the function of mitochondrial proteins. Consequently, the mitochondrial membrane potential, GSH, ROS, and Ca^2+^ levels gradually recover, alleviating the pathological manifestations of ad.

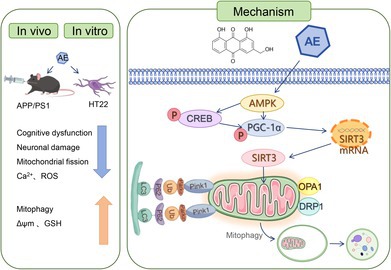

**Abbreviations**
ad
: Alzheimer's disease
AE: Aloe‐emodin
Aβ: β‐Amyloid
MWM: Morris water maze
RAPA: Rapamycin
ROS: Reactive oxygen species
WT: Wild‐type

## 1 Introduction

Alzheimer's disease (ad) has been a common form of aphronesia among older adults, characteristic of progressive memory loss and cognitive decline. Such disease is a category of neurodegenerative disorder that affects the central nervous system [1]. Mitochondria, often referred to as the cell's “powerhouse,” are essential for ATP production, calcium regulation, and managing oxidative stress [2, 3]. In Alzheimer's disease (ad), mitochondrial damage occurs before the onset of related pathology, making it a critical early factor in the disease's progression [4, 5]. Postmortem brain studies of ad patients show mitochondrial dysfunction, with reduced enzyme activity [6] and increased oxidative stress [7]. Dysfunctional mitochondria in neurons lead to lower ATP levels and increased reactive oxygen species (ROS), worsening mitochondrial damage [8]. Mitophagy is a crucial mechanism for mitochondrial quality control that selectively removes damaged or dysfunctional mitochondria to preserve mitochondrial network function and cellular homeostasis [9, 10]. The PINK1‐Parkin pathway serves as a mitophagy pathway that depends on membrane potential [11]. Research suggests that mitochondrial dysfunction occurs before microglial activation and neuroinflammation in ad [12]. Changes in mitochondrial membrane potential, excessive ROS production, and high calcium levels can disrupt mitophagy and impair mitochondrial quality control [13, 14, 15]. When mitophagy is impaired, damaged mitochondria accumulate, leading to increased neuronal injury [16, 17]. Identifying drugs that enhance neuronal mitophagy may help prevent and treat Alzheimer's disease [18]. Indeed, Sirtuin 3 (SIRT3) acts as a histone deacetylase throughout the brain, particularly in the hippocampus [19]. Inhibiting SIRT3 increases mitochondrial ΔΨm and reduces quality, while enhanced SIRT3 expression activates key mitophagy regulators, encompassing Parkin, PINK1, FOXO3a, and the LC3I/LC3II proportion [20, 21]. The AMPK/PGC‐1α/ SIRT3 pathway is believed to have protective actions on oxidative stress and mitochondrial dysfunction [22, 23]. Aloe‐emodin (AE) is an anthraquinone compound present in the roots and rhizomes of traditional Chinese medicinal herbs such as rhubarb, aloe, and cassia seeds [24]. Studies have shown that AE can promote autophagy in tumor cells [25], inhibit the aggregation of ad pathological proteins [26], and exert anti‐inflammatory [27] and antioxidant effects [28]. It is still uncertain whether AE can enhance cognitive function in ad through improved mitophagy.

This study investigates the effects of AE on Alzheimer's disease (ad) using an ad model with APP/PS1 transgenic mice and validation experiments in HT22 cells. From the behavior tests of Morris water maze and Y maze tests, the mice had better performance when treated with AE. Morphological detection of hippocampal neurons found that the mice had less hippocampal damage when treated with AE. Thus, AE improves cognitive dysfunction, reduces hippocampal damage, and alleviates ad symptoms in model mice. Furthermore, we explored the role of mitophagy in both ad and the treatment of AE. We found that AE activates mitophagy in neurons, as the expression of mitophagy‐related protein was demonstrated in immunofluorescence and western blot. We also confirmed that p‐AMPK, PGC‐1α, and SIRT3 were increased expressed when treated with AE. In vitro experiments showed that AE can enhance the mitochondrial function in Alzheimer's disease cell models by promoting mitophagy. AE enhances mitophagy by activating the AMPK/PGC‐1α/SIRT3 pathway. However, when SIRT3 expression is downregulated, mitophagy decreases. Experimental results show that AE can activate mitophagy through this pathway, alleviate cognitive dysfunction in ad, and reduce damage to hippocampal neurons. AE has a neuroprotective effect by addressing mitochondrial dysfunction and is effective in treating ad. This provides modern evidence for the clinical application of traditional Chinese medicine (TCM) in the therapy of ad.

## 2 Materials and Methods

### 2.1 Animal Grouping and Drug Intervention

**FIGURE 1:**
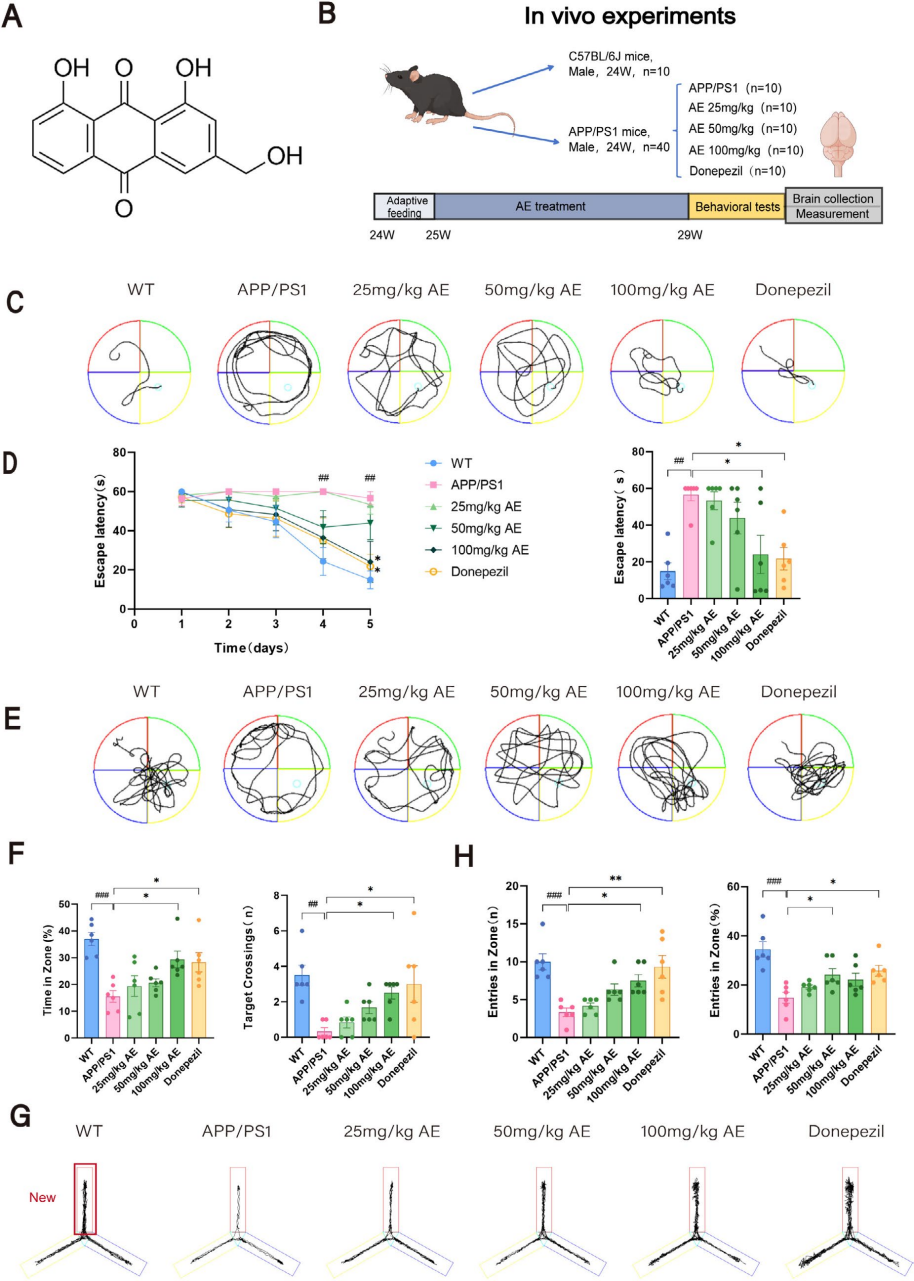
AE improved behavioral performance in the ad mice by the MWM and the Y maze tests. (A) Chemical structure of aloe‐emodin. (B) A diagram illustrating the experimental design for the in vivo studies. (C) Swimming trajectory of mice on the fifth day of the escape latency test. (D) Statistical graph of escape latency. (E) Trajectory diagram of the probe training. (F) Statistics of target quadrant dwell time and crossing times during the probe training. (G) Representative exploration trajectory during the Y‐maze test. The red arm labeled in the figure represents the novel arm. (H) Statistical graph of novel arm entries and the ratio of novel entrance to total entrance. The data from behavioral tests are expressed as mean ± SEM(*n* = 6). ^#^ *p* < 0.05 by contrast to the WT group, *p < 0.05 by contrast to the ad group.

Our study utilized male C57BL/6J mice that were 6 months old, along with APP/PS1 double transgenic mice. The C57BL/6J mice are the wild‐type (WT) group (*n* = 10). The APP/PS1 double transgenic mice were stochastically segregated into the following groups: the Model group, the 25 mg/kg AE group, the 50 mg/kg AE group, the 100 mg/kg AE group [29, 30], and the Donepezil group, with each group consisting of 10 mice (*n* = 10). Following a 1‐week adaptive feed, the mice underwent 28 days of intragastric treatment. The animals were then used for subsequent experiments following the experimental flow chart (Figure 1B).

Aloe‐emodin (Figure 1A), which was purchased from Yuanye Biotechnology Co. Ltd. (Shanghai), was used in the animal experiments with a purity of 97.6%. It was prepared as a suspension in pure water, and the dosage for each group was measured for intragastric administration. Donepezil hydrochloride tablets (from China) were given to the Donepezil group, dissolved in pure water, at a dosage of 2.1 mg/kg via the intragastric route. The WT group received daily intragastric administration of pure water. All procedures undertaken in our research received approvals from the Ethics Committee of the Anhui University of Chinese Medicine (Animal Ethics No.: AHUCM‐mouse‐2024051).

### 2.2 Cell Culture and Drug Treatment

The cells were seeded into culture dishes and incubated in a 37°C, 5% CO_2_ incubator. After 24 h of seeding, the medium was changed, and the Model group was processed using 30 μM Aβ_25‐35_. The AE groups were processed using 30 μM Aβ_25‐35_ [31] and 2, 4, or 6 μM AE, and the RAPA group was processed using 30 μM Aβ_25‐35_ and 200 nM Rapamycin. After 24 h of treatment, various detection experiments were performed.

1 mg of Aβ_25‐35_ was dissolved in 1 mL of deionized water as a stock solution, incubated in a 37°C incubator for 5 days to induce oligomer formation. It was aliquoted into 100 μL volumes and stored at −20°C. A concentration of 30 μM was used in this experiment based on previous research. Aloe‐emodin and rapamycin were each dissolved in DMSO to form stock solutions of 50 and 10 mM. The stock solution of Aloe‐emodin was diluted in a complete medium to the required concentration gradient for further use. The stock solution of Rapamycin was diluted twice in a complete medium to a final concentration of 200 nM.

### 2.3 Behavior Test

#### 2.3.1 Morris Water Maze (MWM) Test

The test was implemented to appraise memory function in mice [32, 33]. Training lasted 4 days. Mice were placed in the water from each quadrant in sequence, and the time to find the platform within 60 s was recorded. If no platform was found within 60 s, the mouse was directed to it and placed there briefly. On day 5, the escape latency test was conducted. The time and swimming path were recorded as the mouse entered from the opposite quadrant to find the platform, with a 60‐s limit. On day 6, the probe test was performed. The platform was removed, and the swimming path, duration, and number of crossings in the object quadrant were recorded within 60 s.

#### 2.3.2 Y‐Maze Test

This experiment was executed to appraise the memory and exploratory abilities of mice [34, 35]. The Y‐maze consisted of three arms connected in the middle to form a “Y” shape, with 120° angles between the arms. A camera and software were used to record the test. During the acquisition phase, the novel arm was blocked, allowing the mouse to explore the other two arms for 10 min. During the test phase, all three arms were opened. The mouse was allowed to explore freely for 5 min, and the movement trajectory and number of entries into the novel arm were recorded.

### 2.4 Brain Collection and Tissue Preparation

After the behavioral experiments, the mice underwent anesthesia through an intraperitoneal injection of sodium pentobarbital, followed by cardiac perfusion. The brains were collected and preserved in paraformaldehyde for embedding and subsequent sectioning and staining. Some mice were sacrificed after anesthesia, and the hippocampus was quickly collected on ice for electron microscopy and western blotting.

### 2.5 Hematoxylin and Eosin (HE) Staining

The sliced brain tissue sections were dewaxed, stained with hematoxylin–eosin (Beyotime, C0105S), dehydrated, cleared, and mounted with neutral resin. After preparation, the sections were observed using a slide scanner (Wisleap WS‐10, China).

### 2.6 Transmission Electronic Microscopy

The mice's brains were extracted on ice within 2 min, and the hippocampus was promptly isolated and immersed in glutaraldehyde. After 24 h, the fixative was discarded, and the samples were placed in a PBS buffer for 6 h, followed by fixation in osmium acid. After 30% and 50% ethanol, the samples were stained with 70% ethanol uranyl acetate for 3 h before further dehydration with 80%, 95%, and 100% ethanol. They were then placed in propylene oxide for 2 h, followed by immersion in pure epoxy resin for 3 h. After embedding in purified epoxy resin, the samples were placed in a 45°C oven for 12 h, then in a 72°C oven for 24 h. The embedded blocks were trimmed, cut into ultrathin sections of 70 nm thickness, stained with lead, and observed using transmission electron microscopy.

### 2.7 Immunohistochemistry

The sections were subjected to xylene, 100%, 95%, and 80% ethanol, washed with running tap water, and then high‐pressure antigen retrieval was performed. Two liters of citrate buffer antigen retrieval solution were prepared, and the sections were placed in a pressure cooker, boiled, and heat‐repaired. The tissues were circled with an immunohistochemical pen and placed in an incubation box, and 3% H_2_O_2_ was added to the tissue and incubated. After washing with PBS, the slides were dried, and primary antibodies Opa1 (Zenbio), Drp1 (Proteintech), P62 (Servicebio), and PINK1 (Beyotime) were added, incubating at 37°C. Then, it was replaced with the secondary antibody. After washing with PBS, DAB chromogen was added, and the staining process was monitored under a microscope, stopping the reaction when positive staining was observed. The sections were stained with hematoxylin, then differentiated with hydrochloric acid alcohol, blued with lithium carbonate, dehydrated, cleared, and mounted. The sections were observed using a slide scanner (Wisleap WS‐10, China).

### 2.8 Immunofluorescence

For the animal immunofluorescence experiment, first, the paraffin sections were dewaxed to water. After washing with PBS, the sections were placed into citrate buffer and microwaved for 15 min. After cooling, it was washed with PBS. Then goat serum‐blocking solution was added and incubated in a wet box for 45 min. Diluted primary antibodies LC3B (Zenbio), Parkin (Zenbio), SIRT3 (Affinity), and p‐AMPK (Beyotime) were added and nurtured at 4°C. The next day, fluorescent secondary antibodies were added and incubated, followed by DAPI staining. After washing, an anti‐fluorescence quenching agent was applied to cover the section.

In the cellular fluorescence experiment, the treated HT22 cells are fixed with paraformaldehyde. Then, the cells were blocked for 60 min using an immunostaining blocking solution (Beyotime; P0102) and permeabilized with an immunostaining permeabilization solution (Beyotime; P0096) for 10 min. Primary antibodies LC3B (Zenbio), p‐AMPK (Beyotime), AMPK (Beyotime), Parkin (Zenbio), and PINK1 (Beyotime) were placed in the wells and cultivated at 4°C. The next day, the cells were fostered with the Cy3‐labeled donkey anti‐rabbit IgG (Servicebio) and Alexa Fluor 488‐labeled goat anti‐rabbit IgG (Servicebio). After washing, the cells were stained with DAPI and mounted using an anti‐fluorescence quenching mounting medium (Beyotime, P0131). The fluorescence was observed using a fluorescence or confocal laser scanning microscope (Olympus IX‐81, Olympus FV1000, Japan).

### 2.9 CCK‐8 Cell Viability Assay

To determine AE's safe concentration range and optimal dosing concentration, HT22 cells were placed within 96‐well plates. After incubating for 1 day, the cells adhered and extended synapses. Moreover, they were modeled through 30 μM Aβ_25‐35_ and processed using discrepant concentrations of AE. After 1 day of intervention, CCK‐8 solution (Beyotime, C0041) was added. Absorbance readings were taken with a multifunctional enzyme reader (Promege 1500, USA).

### 2.10 EDU Assay

The EDU assay detected DNA synthesis within 3 h, using the cell proliferation assay kit (Beyotime, C0075S) [36]. After HT22 cells in 96‐well plates were treated, the EDU working solution was added. After EDU labeling, the fixative (Beyotime, P0098) was added. After washing, 100 μL of permeabilization buffer (Beyotime, P0096) was added and incubated for 10–15 min. After washing, 0.5 μL of Click reaction solution was added and incubated in the dark. After staining the nuclei with Hoechst 33342, the fluorescence was observed under a microscope (IX‐81, Olympus, Japan).

### 2.11 Mitochondrial Membrane Potential (Δψm) Measurement

The enhanced Δψm detection kit (JC‐1, Beyotime, C2003S) was used [37]. HT22 cells in 96‐well plates were washed twice with PBS, and JC‐1 dyeing solution was supplied. The cells were fostered for 20 min in an incubator. Finally, the cells were examined at 200× magnification using a fluorescence microscope.

### 2.12 ROS Measurement

The ROS detection kit (Beyotime, S0033S) was applied to determine ROS levels in each group of cells [38]. The cells were cultivated through the diluted DCFH‐DA at 37°C for 40 min, with the medium in 96‐well plates eliminated. Such cells were rinsed and examined through a fluorescence microscope at 200× magnification.

### 2.13 Ca^2+^ Measurement

Fluo‐4 AM (Beyotime, S1061S) measured Ca^2+^ levels [39]. The medium was removed from the HT22 cells. The cells were cultured at 37°C for 30 min in an incubator with 100 μL of Fluo‐4 staining solution poured into each well. After incubation, the cells were examined and imaged under a fluorescence microscope.

### 2.14 Mitochondrial Superoxide and GSH Level Measurement

The mitochondrial superoxide detection kit (MitoSOX Red, Beyotime, S0061S) was used. After digesting and collecting the cells, the cells were resuspended in a MitoSOX Red staining solution. The cells were cultured at 37°C for 30 min and centrifuged. After washing, the cells were resuspended in an appropriate volume of PBS and analyzed using flow cytometry. The GSH and GSSG detection kit (Beyotime, S0053) [40] was used to measure GSH levels in HT22 cells after drug treatment. Absorbance was measured using a multifunctional enzyme reader (Promege 1500, USA).

### 2.15 Western Blotting

After drug treatment, HT22 cells were collected using Western and IP cell lysis buffer (Biosharp; BL509A) containing PMSF (Biosharp; BL507A), followed by sonication on ice for 3 min. The lysates underwent centrifugation at 12,000 × g for 5 min at 4°C, with the supernatant collected. After electrophoresis and membrane transfer, it was blocked with skimmed milk. Then, the PVDF membrane was placed into the primary antibody solution and incubated at 4°C: Drp1 (Proteintech), Opa1 (Zenbio), P62 (Servicebio), LC3B (Zenbio), Beclin1 (Zenbio), p‐AMPK (Beyotime), AMPK (Beyotime), PGC‐1α (Zenbio), SIRT3 (Affinity), β‐actin (Proteintech), and GAPDH (Proteintech). After the primary antibody incubation, it was washed with TBST. It was incubated with the corresponding secondary antibody (Proteintech) at normal temperature. After rinsing through the TBST, the developer solution was added evenly, and the blots were imaged through a gel imaging mechanism. Band intensity was analyzed using ImageJ software.

### 2.16 Transfection

The most efficient transfection sequence was selected from three designed sequences. HT22 cells were divided into five groups: Blank, NC, mSIRT3‐siRNA‐1, mSIRT3‐siRNA‐2, and mSIRT3‐siRNA‐3. After 1 day of growth, the cells were transfected using the prepared transfection reagent (CALNP, DN001) for 24 h. The NC group was processed through the identical volume of transfection reagent with no transfection sequence. After transfection, the cells were subjected to modeling and drug treatment. After 24 h, RNAs were extracted through a total RNA extraction kit (CALNP, FZ007), and SYBR Green RT‐qPCR was performed using a one‐step RT‐qPCR kit (CALNP, FZ005). The subsequent steps were performed using an RT‐PCR machine (LightCycler 96, Roche, Switzerland). SIRT3 primer sequences and transfection sequences are shown in Figure 7A.

### 2.17 Statistical Analysis

All statistical analyses were performed using SPSS (v 26.0) and GraphPad Prism (v 8.0). Data were presented as mean ± standard error of the mean (SEM), and a *p* < 0.05 was considered statistically significant. Normality of data distribution was assessed by the Shapiro–Wilk test, and homogeneity of variances was verified using Levene's test. For data conforming to both normality and homogeneity of variances, one‐way analysis of variance (ANOVA) was applied for intergroup comparisons. If the normality assumption was violated, nonparametric tests were employed: the Mann–Whitney test for comparisons between two groups and the Kruskal–Wallis test for comparisons among more than two groups.

## 3 Results

### 3.1 AE Alleviates Cognitive Dysfunction in AD Model Mice

We evaluated the effect of AE on cognitive dysfunction in ad mice using the MWM and Y‐maze tests after 28 days of oral administration. As shown in Figure 1C, the mice underwent escape latency training for the first 5 days. On day 5, the ad mice spent more time to position the platform compared to the WT mice (*p* < 0.01). However, treatment with either AE or donepezil in ad mice significantly reduced the time spent to discover the platform (*p* < 0.05), as illustrated in Figure 1D. Furthermore, during the probe trial (Figure 1E), the percentage of time spent in the object quadrant was remarkably decreased among ad mice (*p* < 0.001), and the quantity of crossings into the object quadrant (*p* < 0.01) was in marked contrast with the WT group (Figure 1F). These deficits improved with AE and donepezil treatment, with the 100 mg/kg dose of AE demonstrating the most significant effect (*p* < 0.05). To assess short‐term working memory, we performed the Y‐maze test (Figure 1G). The ad mice made significantly fewer entries into the novel arm than WT (*p* < 0.001) and drug‐treated ad animals (*p* < 0.05) as shown in Figure 1H. This indicates that treatment with AE improved the impairment of short‐term working memory observed in ad mice.

### 3.2 AE Mitigates Pathological Features in AD Model Mice

**FIGURE 2:**
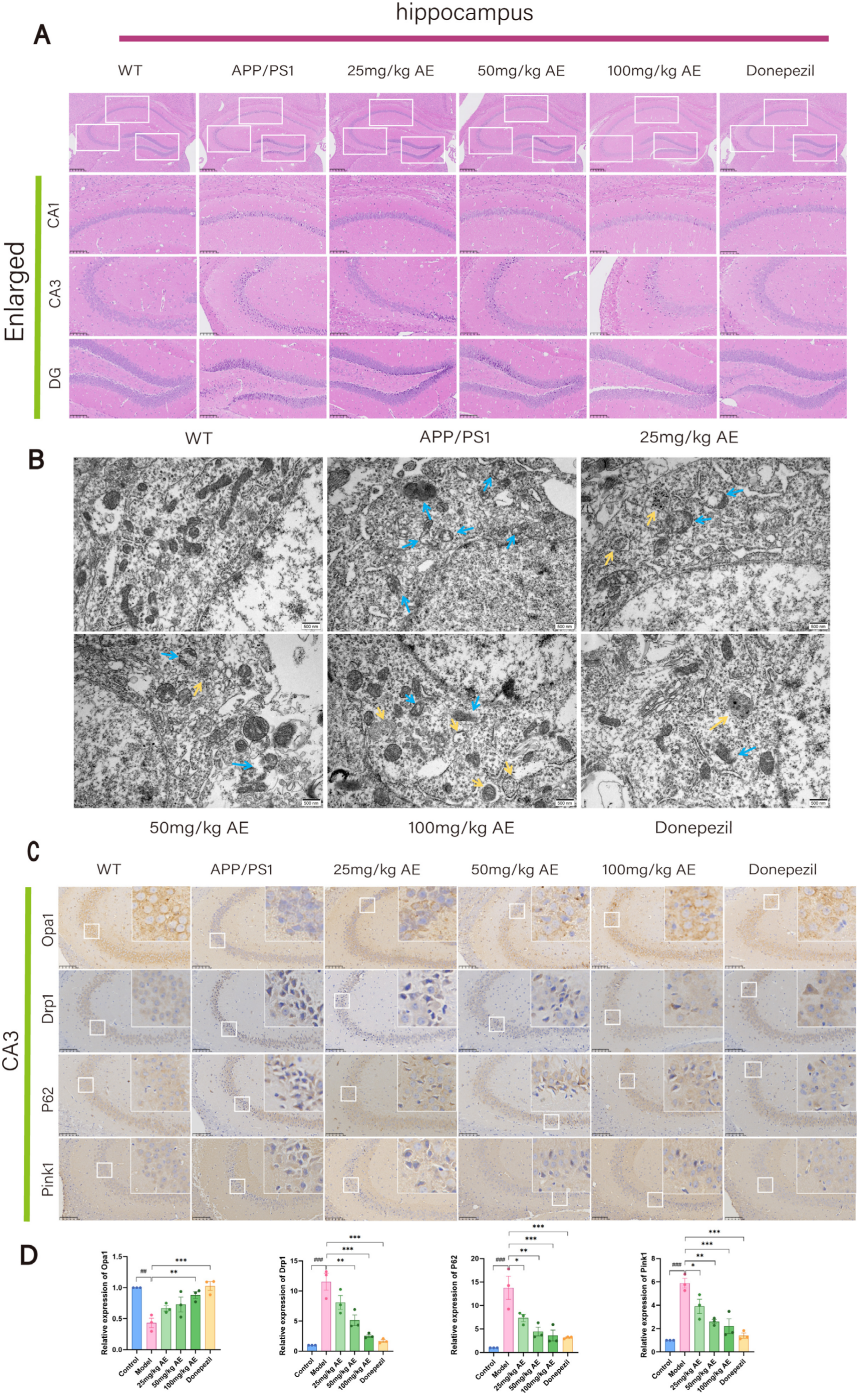
AE protects hippocampal neurons in ad mice. (A) Hematoxylin and Eosin (H&E) staining of the mouse hippocampus. Scale bar = 100 μm. (B) Damaged mitochondria and mitophagy in neurons of the mouse hippocampus were visualized using transmission electron microscopy. Blue arrows highlight damaged mitochondria, while yellow arrows point to autophagosomes and autolysosomes. Scale bar = 500 nm. (C) Immunohistochemical (IHC) detection of mitochondrial fission, fusion, and mitophagy‐related protein expression in different groups. Expression of Opa1, Drp1, P62, and Pink1 in mouse hippocampal neurons. Scale bar = 100 μm. (D) Statistical evaluation of IHC findings. The outcomes were denoted as mean ± SEM(*n* = 3). ^#^ *p* < 0.05 in contrast with the WT group, *p < 0.05 in contrast with the ad group.

Alzheimer's disease is strongly linked to damage in the neurons of the hippocampus. HE staining analysis of the hippocampal CA1, CA3, and DG areas among mice revealed that neurons within the wild‐type (WT) group showed normal morphology (Figure 2A). In contrast, neurons within the APP/PS1 group showed signs of atrophy, disorganized arrangement, hyperchromatic staining, and deformed cell bodies. The AE and donepezil‐treated groups had a more uniform cell distribution than ad mice, indicating significant recovery from neuronal damage. Among the AE treatment groups (25, 50, and 100 mg/kg), the group receiving 100 mg/kg showed the most significant improvement. Transmission electron microscopy was applied to check the intracellular organelle of hippocampal neurons. Compared to the WT group, ad mice displayed swollen, ruptured mitochondria, disrupted cristae, absent outer membranes, and reduced mitophagy (Figure 2B). Autophagosomes and autolysosomes were observed in the AE and donepezil groups, showing damaged mitochondria being engulfed, which indicate activation of mitophagy.

### 3.3 AE Activates Mitophagy in Neurons to Alleviate AD Symptoms

**FIGURE 3:**
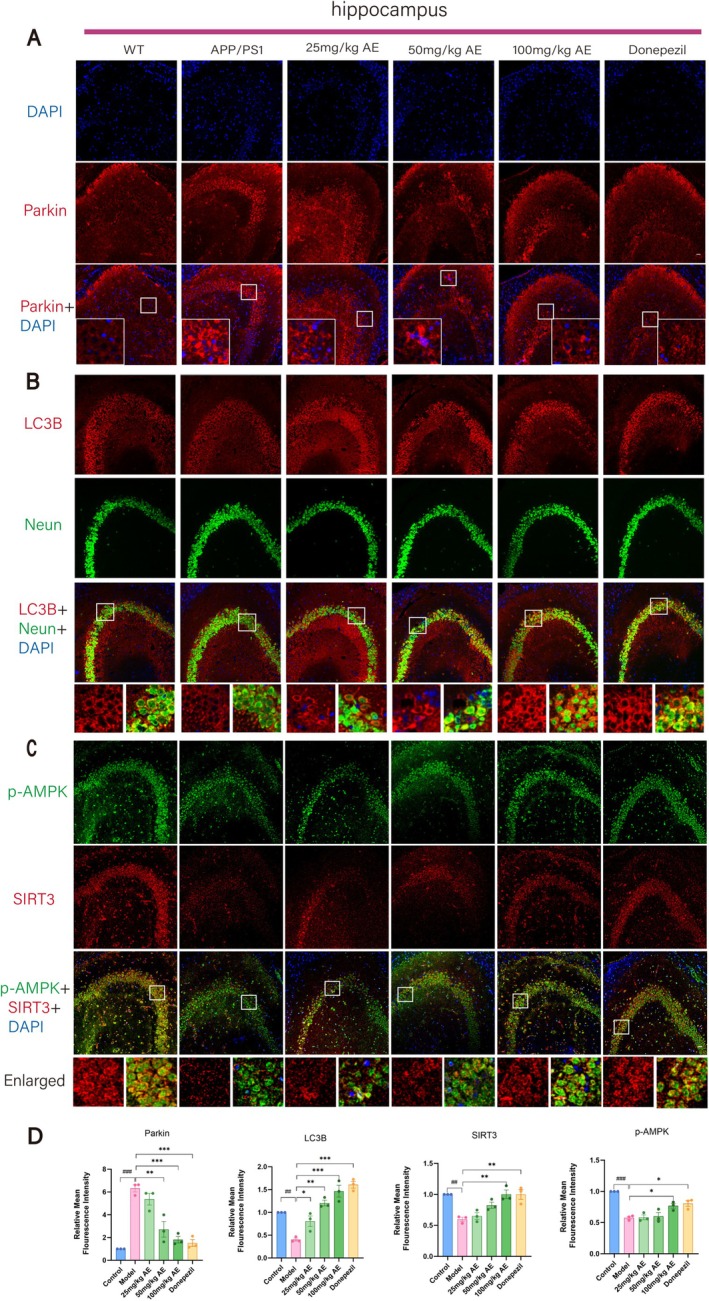
Aloe‐emodin activates mitophagy in ad mice neurons. (A) Fluorescence staining of Parkin (red) in the mouse hippocampus. The cell nuclei were stained with DAPI (blue). (B) Double fluorescence staining of LC3B (red) and Neun (green) in the mouse hippocampus. (C) Double fluorescence staining of p‐AMPK (green) and SIRT3 (red) among mouse hippocampus. Scale bar = 20 μm. (D) Statistical analysis of the IF results. The outcomes were denoted as mean ± SEM(*n* = 3). ^#^ *p* < 0.05 in comparison with the WT group, *p < 0.05 in comparison with the ad group.

The expression of mitochondrial fission/fusion proteins (OPA1 and DRP1) and mitophagy markers (PINK1, Parkin, P62, Beclin1, and LC3B) in Alzheimer's mouse hippocampal neurons was analyzed by IHC and IF staining (Figures 2C and 3A). Opa1 expression in neurons of ad mice was decreased remarkably (*p* < 0.01) in comparison with the WT group but increased after treatment. Opa1 expression increased in the 25, 50, and 100 mg/kg AE groups, and the donepezil group showed the most significant effect (*p* < 0.05) (Figure 2D). In ad mice, neuron levels of DRP1, P62, Pink1, and Parkin exceeded prominently those in the WT group (*p* < 0.001). After administering AE and donepezil, expression levels significantly decreased (*p* < 0.05), most notably in the 100 mg/kg AE and donepezil groups (Figures 2D and 3D). LC3B levels were low in WT and ad mice but increased after AE and donepezil treatment (*p* < 0.05). It is worth noting that the moderate dose of 50 mg/kg also significantly regulated the expression of DRP1, P62, LC3B, pink1, and Parkin (*p* < 0.05), showing a dose‐dependent effect.

Consequently, AE improved mitophagy in damaged neurons of ad mice, enhanced mitochondrial fusion, and optimized mitochondrial fission (Figure 3B,D). p‐AMPK and SIRT3 play important roles in regulating mitochondrial function, energy metabolism, autophagy, and mitophagy. The p‐AMPK and SIRT3 expressions were analyzed using immunofluorescent (IF) double staining (Figure 3C). As illustrated in Figure 3D, the p‐AMPK and SIRT3 levels in Alzheimer's disease (ad) mice were much lower than those in the WT group (*p* < 0.01). However, the reductions were significantly mitigated following treatment with 100 mg/kg of AE and donepezil (*p* < 0.05).

### 3.4 AE Promotes Proliferation of AD Model Cells

**FIGURE 4:**
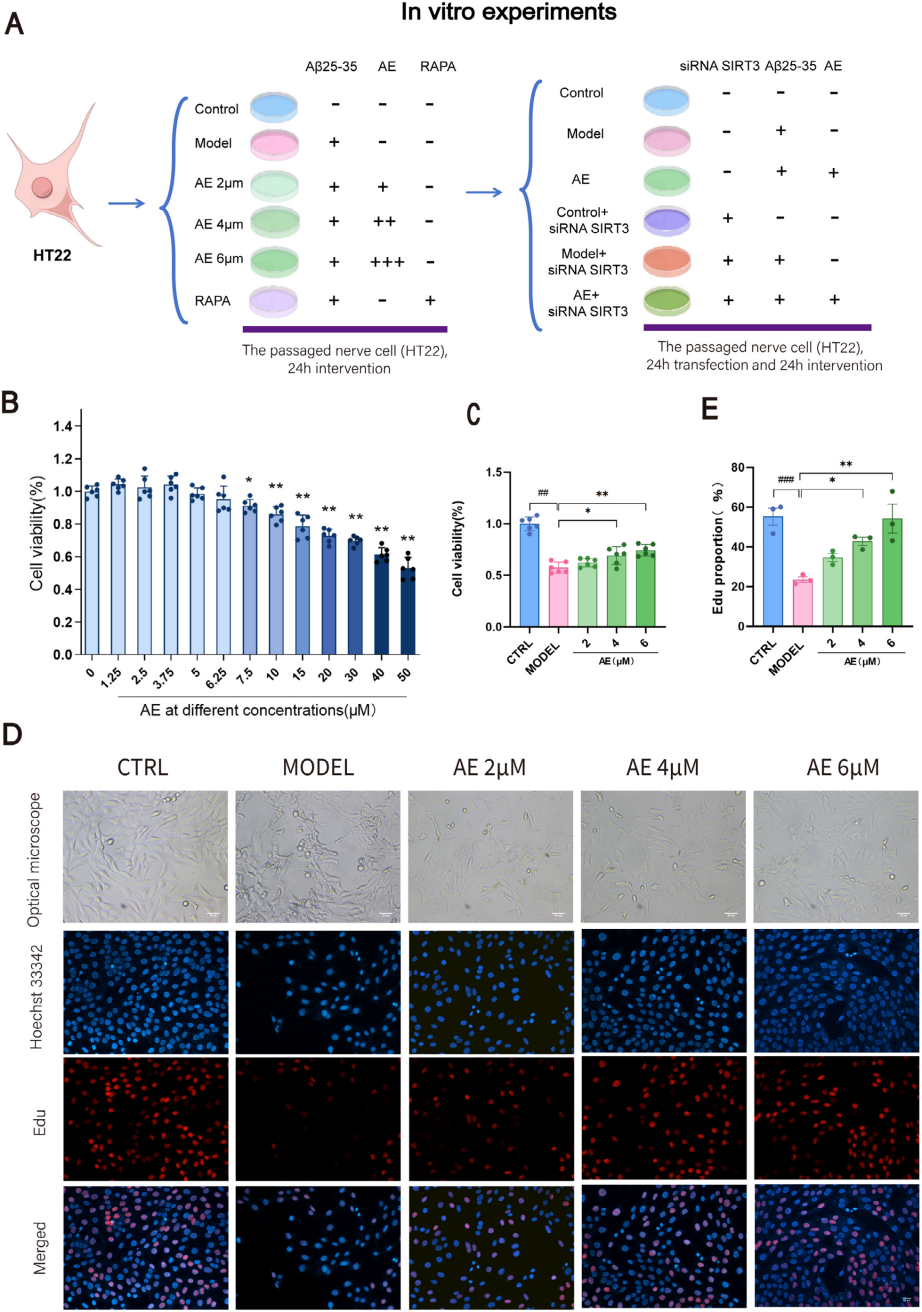
AE Promotes Proliferation of ad Model Cells. (A) HT22 treatment in each group. (B) Effect of different concentrations of AE on HT22 cell viability. (C) Screening of the optimal AE treatment concentration. (D) Light microscopy images of cells treated with 2, 4, and 6 μM AE, along with EDU (red) assay images. The cell nuclei were stained with Hoechst 33342 (blue). Scale bar = 20 μm. (E) Statistical analysis on EDU assay results. The outcomes were denoted as mean ± SEM(*n* = 3). ^#^ *p* < 0.05 by comparison with the WT group, *p < 0.05 by comparison with the ad group.

The workflow for neuronal cell processing is shown in Figure 4A. We conducted a CCK‐8 assay on HT22 cells post‐Alzheimer's disease modeling to determine the optimal concentrations of AE. At 7.5 μM AE, cell viability significantly decreased (*p* < 0.05), and at 50 μM, it dropped by nearly 50% (*p* < 0.01) (Figure 4B). We chose concentrations of 2, 4, and 6 μM to determine the optimal treatment level. As illustrated in Figures 4C and 6 μM AE exhibited the most significant protective effect on ad‐modeled HT22 cells (*p* < 0.01). The cell morphology observed using an optical microscope is illustrated in Figure 4D. In contrast to the control group, the model group showed a reduction in cell count, with cells appearing shrunken and exhibiting broken synapses. Treatment groups with 2, 4, and 6 μM AE had better cell conditions, with fewer shrunken cells and improved intercellular connections. We conducted an EDU assay to observe DNA synthesis over 3 h, as shown in Figure 4D,E. The Model group exhibited a marked reduction in DNA synthesis by contrast with the controls (*p* < 0.01). After therapy, DNA synthesis gradually rose, with the 6 μM AE group showing the most significant rise (*p* < 0.01). Thus, AE demonstrates protective effects on ad‐model cells and encourages their proliferation.

### 3.5 AE Improves Mitochondrial Function in AD Cell Models

**FIGURE 5:**
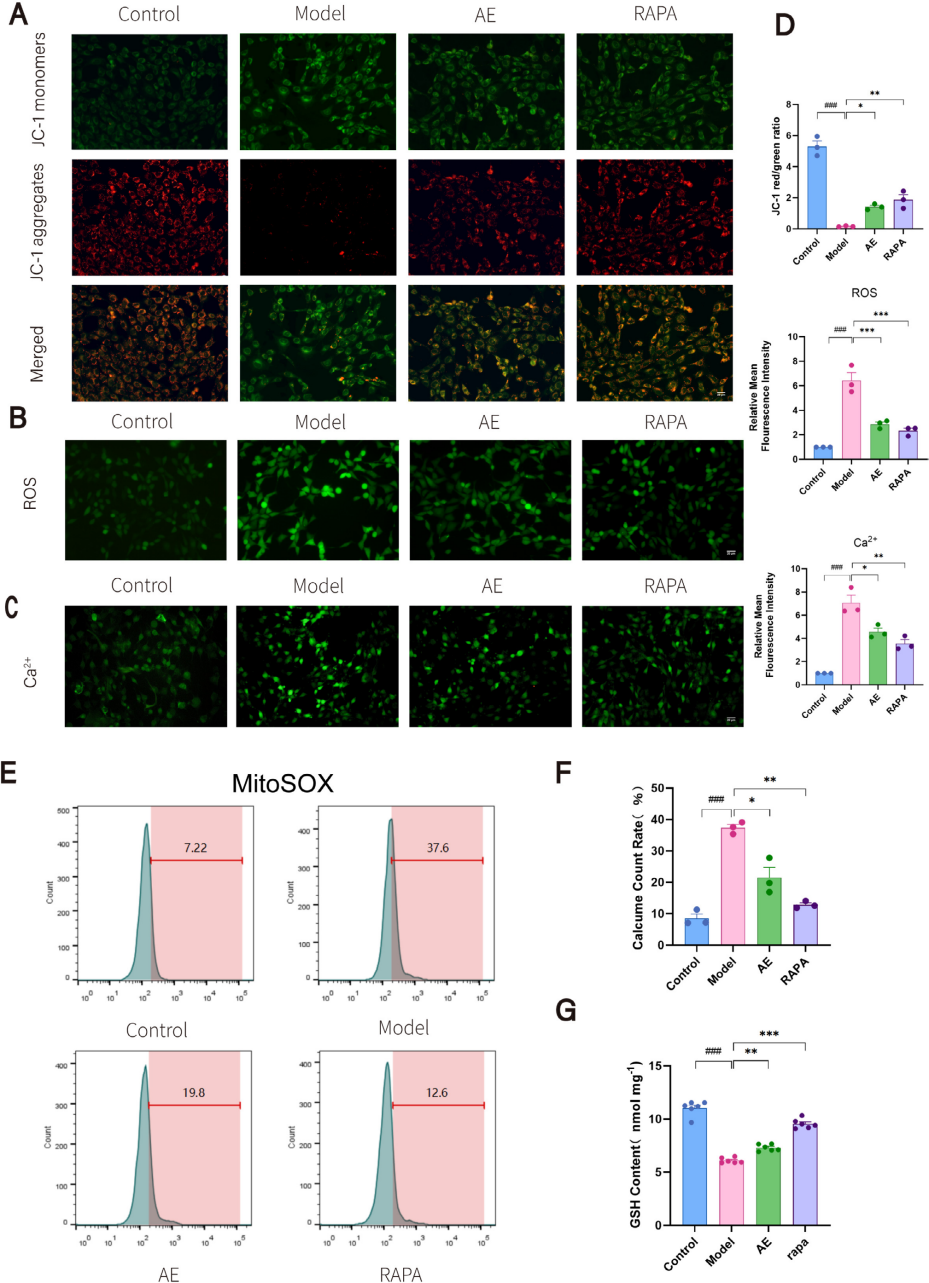
AE improves mitochondrial function in ad cell models. (A) Fluorescence images of JC‐1 staining in HT22 cells. (B) Fluorescence images of ROS detection. Red fluorescence indicates high mitochondrial membrane potential (aggregated JC‐1), green fluorescence represents low mitochondrial membrane potential (monomeric JC‐1). (C) Fluorescence images of Ca^2+^ measurement. (D) Statistical analysis of JC‐1, ROS, and Ca^2+^ results. (E) Flow cytometry results for mitochondrial superoxide detection (MitoSOX). (F) Statistical analysis of MitoSOX results. (G) Statistical analysis of GSH levels. Scale bar = 20 μm. The outcomes were denoted as mean ± SEM(*n* = 3). ^#^ *p* < 0.05 in contrast with the WT group, *p < 0.05 in contrast with the ad group.

We evaluated mitochondrial function in HT22 cells by measuring Δψm (Figure 5A,D), ROS (Figure 5B,D), Ca^2+^ (Figure 5C,D), mitochondrial superoxide (Figure 5E,F), and GSH levels (Figure 5G). HT22 cells processed using the Aβ_25‐35_ exhibited reduced Δψm and GSH levels and increased ROS, Ca^2+^, and superoxide compared to the controls (*p* < 0.01). Consequently, this indicated significant mitochondrial damage and dysfunction associated with ad progression. In contrast, the AE and RAPA groups demonstrated trends that were markedly different from those observed in the model group (*p* < 0.05). These groups showed improvements in mitochondrial function, although the levels did not return to normal. These findings indicate that AE enhances mitochondrial function in ad model cells, with autophagy potentially contributing to mitochondrial protection.

### 3.6 AE Activates Mitophagy and Attenuates AD Progression via the AMPK/PGC‐1α/SIRT3 Pathway

**FIGURE 6:**
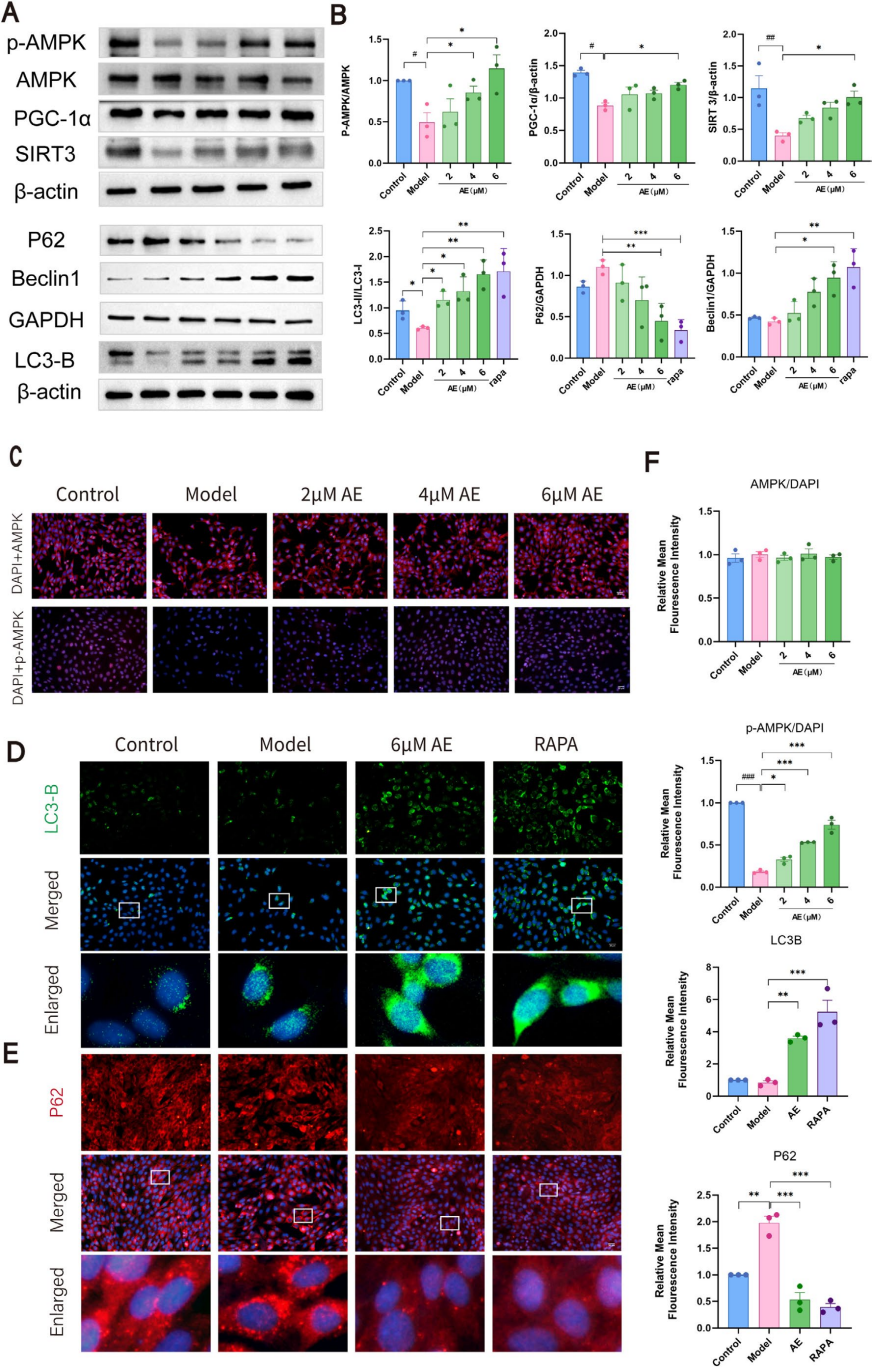
AE treatment modulates mitophagy and proteins related to the AMPK/PGC‐1α/SIRT3 pathway. (A) Western blot results of AMPK, p‐AMPK, PGC‐1α, SIRT3, and mitophagy‐related proteins (LC3B, P62, Beclin1) in HT22 cells. (B) Statistical analysis of the western blotting results. (C) Fluorescence staining of p‐AMPK, AMPK (red), and DAPI (blue) among HT22 cells. (D) Fluorescence staining of LC3B (green) and DAPI (blue) among the ditto cells. (E) Fluorescence staining of P62 (red) and DAPI (blue) among the ditto cells. Scale bar = 20 μm. (F) Statistical analysis on the IF results. The outcomes were denoted as mean ± SEM(*n* = 3). ^#^ *p* < 0.05 by contrast with the WT group, *p < 0.05 by contrast with the ad group.

Levels of p‐AMPK, AMPK, PGC‐1α, SIRT3, and mitophagy markers were assessed by western blotting (Figure 6A,B) and immunofluorescence (Figure 6C–E). The model group had lower PGC‐1α, p‐AMPK, and SIRT3 levels than the controls (*p* < 0.05), alongside a slight decline in mitophagy. AE treatment significantly increased Beclin1, LC3B, PGC‐1α, p‐AMPK, and SIRT3 (*p* < 0.05) while decreasing P62 (*p* < 0.05), indicating enhanced mitophagy levels.

### 3.7 The Knockdown of SIRT3 Increased Mitochondrial Damage and Reduced Mitophagy in HT22 Cells

**FIGURE 7:**
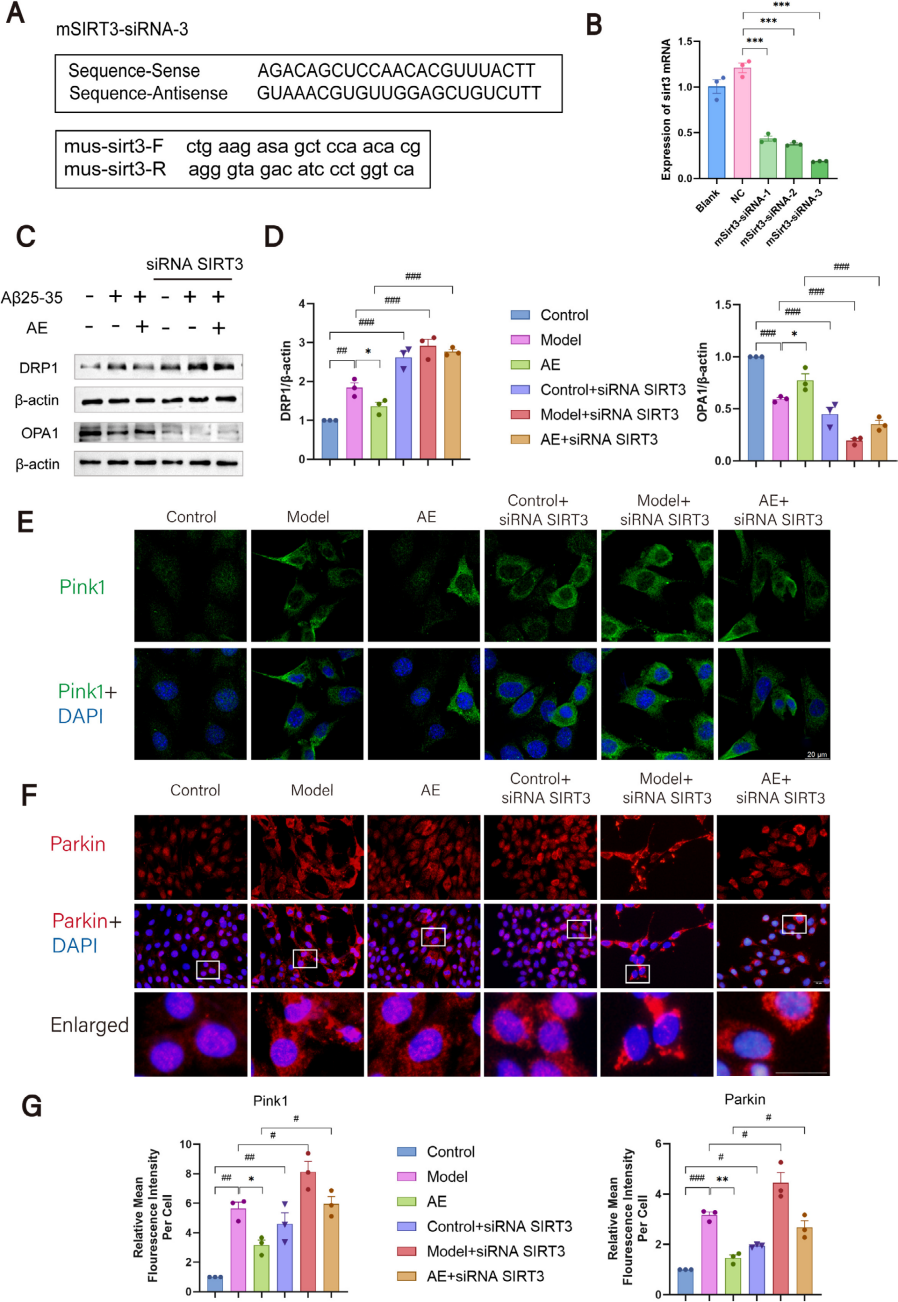
Changes in mitochondrial fission, fusion, and mitophagy‐related proteins after SIRT3 siRNA transfection. (A) SIRT3 primer sequences and SIRT3 siRNA transfection sequence. (B) RT‐qPCR detection of transfection efficiency for the three sequences. (C) Western blot results of DRP and OPA1. (D) Statistical analysis of the western blot results. (E) Fluorescence staining of Pink1 (green) and DAPI (blue) among HT22 cells. (F) Fluorescence staining of Parkin (green) and DAPI (blue) among the ditto cells. Scale bar = 20 μm. (G) Statistical analysis on the IF results. The outcomes were denoted as mean ± SEM(*n* = 3). ^#^ *p* < 0.05 in contrast with the WT group, *p < 0.05 in contrast with the ad group.

To study SIRT3's effects on mitochondrial dynamics and mitophagy in HT22 cells treated with Aβ_25‐35_ and AE, we transfected the cells with SIRT3 siRNA. We then employed western blotting and immunofluorescence to observe changes in these proteins. RT‐qPCR was utilized to identify the most effective transfection sequence, as shown in Figure 7A,B. The results showed that sequence 3 demonstrated the highest transfection efficiency (*p* < 0.001), and this sequence was chosen for future tests. Western blotting evaluated fusion proteins and mitochondrial fission among the Control, Model, and AE groups after SIRT3 siRNA transfection (Figure 7C,D). Before transfection, the Model group had higher DRP1 expression (*p* < 0.01) and lower OPA1 expression (*p* < 0.001) than the controls. After AE treatment, DRP1 decreased and OPA1 increased in contrast to the Model group (*p* < 0.05). After transfection, DRP1 levels rose (*p* < 0.001), while OPA1 levels decreased (*p* < 0.001) in all groups.

Immunofluorescence was utilized to observe changes in mitophagy‐related proteins (Pink1, Parkin) following SIRT3 siRNA transfection, as shown in Figure 7E,F. The analysis indicated that the expressions of Pink1 and Parkin in the Model group remarkably surpassed those in the controls (*p* < 0.001). After treatment, the gene expressions were lower than those in the Model group (*p* < 0.05). Pink1 and Parkin levels in the total transfected groups exceeded those in the non‐transfected Control, Model, and AE groups (*p* < 0.05) (Figure 7G). These findings indicate that mitochondrial damage is increased and mitophagy is decreased in HT22 cells transfected with SIRT3 siRNA. SIRT3 is an important protective player in sustaining mitochondrial function among cells modeled for Alzheimer's disease.

## 4 Discussion

**FIGURE 8:**
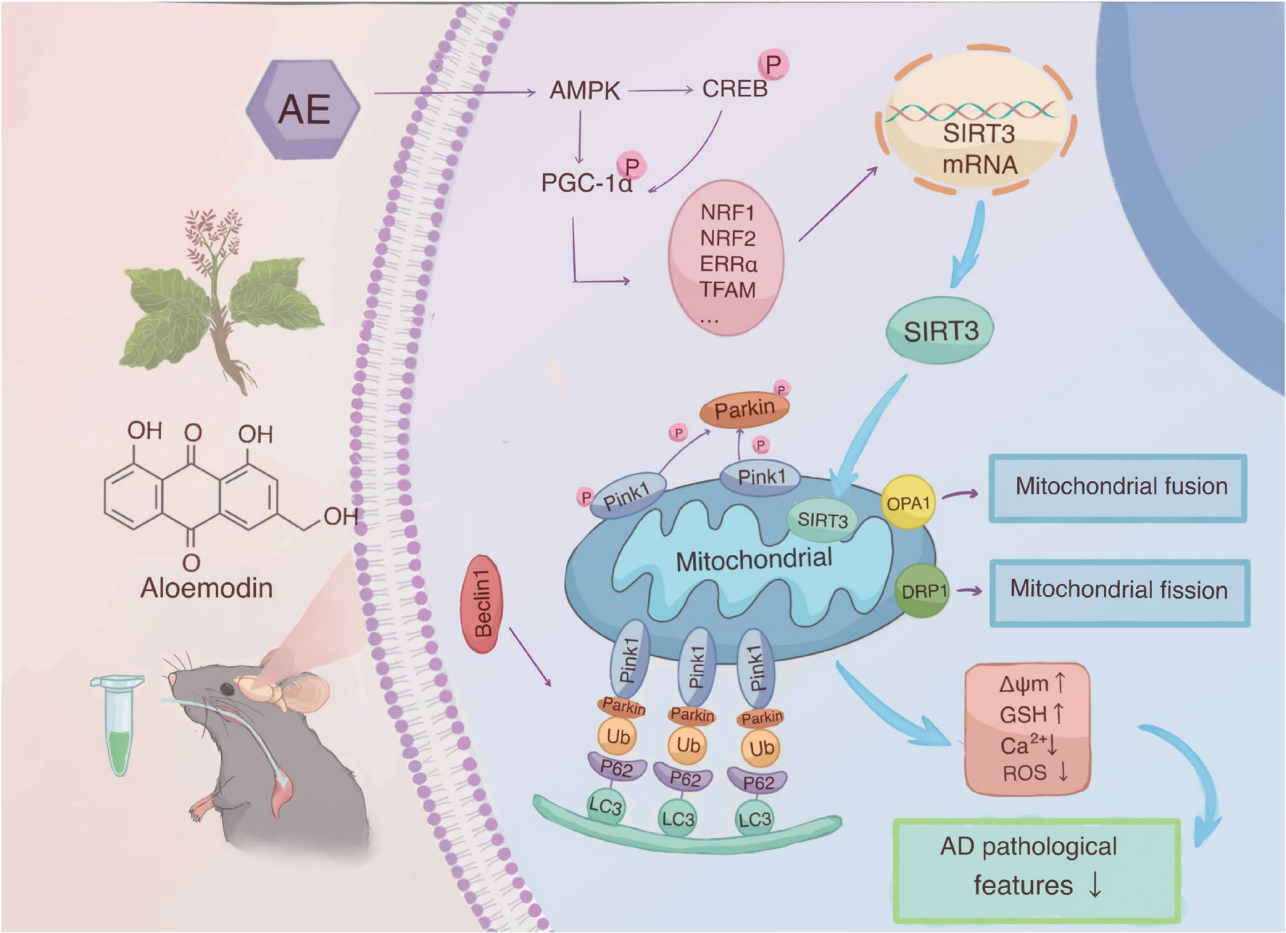
The illustration of the underlying mechanism through which aloe‐emodin induces mitophagy by means of the AMPK/PGC‐1α/SIRT3 pathway within an Alzheimer's disease (ad) model.

AD becomes an extensive dementia, representing 60%–80% of the whole dementia patients [41]. Neuronal damage is usually irreversible, with very limited repair capabilities. This is why most medications aim to slow down neurodegenerative processes and delay cognitive decline [42]. The therapeutic effect of aloe‐emodin (AE), as utilized in this study, is to slow the ad progression rather than reverse it. Among APP/PS1 double transgenic mice, amyloid‐beta (Aβ) deposition in the brain begins to gradually appear around 4–6 months of age, peaking at 9–12 months [43]. We used 6‐month‐old mice and collected samples at 7.5 months to investigate the neuroprotective effects of AE. The study found that AE alleviates cognitive dysfunction and mitigates pathological features in an ad model. AE promotes cell proliferation and enhances mitochondrial function in models of Alzheimer's disease (ad). When mitophagy is enhanced, the expression of Beclin1, LC3, P62, Parkin, and PINK1‐related proteins changes. Consequently, mitochondrial membrane potential, GSH, ROS and Ca^2+^ levels gradually recover, alleviating the pathological manifestations of ad (Figure 8). Additionally, knocking down SIRT3 leads to increased mitochondrial damage and a reduction in mitophagy in HT22 cells. Aloe‐emodin enhances SIRT3 expression through such a pathway, protecting hippocampal neurons among Alzheimer's disease mice.

Our experiment confirmed that AMPK was activated, while other studies on AE found that ATP significantly increased after AE administration [44]. This suggests that AE may activate AMPK independently of the AMP/ATP and ADP/ATP ratio mechanism. Additionally, our research and other studies on AE show that after administration, ROS expression levels decrease and mTOR expression levels increase [45]. In conclusion, we speculate that aloe‐emodin may activate AMPK through upstream kinase pathways (such as CaMKKβ or LKB1) [46, 47] and inhibit negative regulatory pathways. This leads to the generation of biologically active p‐AMPK, which then enhances PGC‐1α activity through direct phosphorylation and synergistic deacetylation. AMPK can enhance the transcriptional activity of PGC‐1α by phosphorylating multiple sites (Thr177/Ser538), promoting its binding to nuclear receptors. Additionally, through the AMPK‐SIRT1‐PGC‐1α axis, it could synergistically enhance mitochondrial function. PGC‐1α interacts with nuclear respiratory factors (Nrf‐1 and Nrf‐2), directly promoting the transcription of the SIRT3 gene. Increased expression of SIRT3 in mitochondria promotes mitophagy and regulates the function of mitochondrial proteins.

Mitophagy is a crucial component of the mitochondrial quality control system. It is a specific type of autophagy that targets and eliminates damaged or dysfunctional mitochondria [48]. Damaged mitochondria are selectively removed by autophagy to maintain mitochondrial function and cell homeostasis. The PINK1‐Parkin pathway is an autophagy pathway that relies on the membrane potential of mitochondria [49]. When mitochondria become damaged, the inner mitochondrial membrane continues to depolarize, causing PINK1 to accumulate in the outer mitochondrial membrane [50]. Then, PINK1 activates and recruits Parkin to the damaged mitochondria [51], which ubiquitinates the mitochondrial external membrane proteins MFN1/2 and VDAC1. For example, OPTN, p62, NDP52, and NBR1 gather on the mitochondrial external membrane. Such a gathering causes recruitment of ubiquitinated products to autophagosomes, in conjunction with LC3. Subsequently, mature autophagosomes fuse with lysosomes, allowing for the degradation of autophagic contents by the lysosomes. Our study found that, compared to the control group, increased neuronal damage correlates with higher expression levels of PINK1 and Parkin. AE treatment lowered their levels. In mitophagy, Beclin1 acts early, regulating the formation and maturation of autophagosomes [52, 53]. LC3‐I is subsequently converted into LC3‐II in the cytoplasm [54], and P62 recognizes ubiquitinated mitochondria and binds to LC3‐II, facilitating their encapsulation into autophagosomes [55]. Our findings showed that the level of mitophagy‐related proteins was decreased within the ad model group. However, following treatment with AE and donepezil, mitophagy levels increased, resulting in the restoration of mitochondrial function.

SIRT3 is a vital mitochondrial deacetylase that regulates various cellular functions by deacetylating mitochondrial proteins, including the induction of autophagy, particularly mitophagy [56, 57]. SIRT3 expression is reduced in ad [56, 58], and activating SIRT3 is a potential therapeutic approach for neurodegenerative diseases [59]. Some studies have shown that enhancing the expression or activity of SIRT3 can improve mitophagy in APP/PS1 transgenic mice [60], alleviating synaptic damage and cognitive deficits [61, 62]. The activity and expression of peroxisome proliferator‐activated receptor gamma coactivator 1‐alpha (PGC‐1α) correlate with SIRT3 expression [63]. PGC‐1α indirectly controls SIRT3 transcription by promoting NRF2 [64] and ERRα [63]. AMPK targets several cellular proteins, such as cAMP response element‐binding protein (CREB) as a famous catalyst of PGC‐1α can increase mitochondrial SIRT3 expression [65]. AMPK, PGC‐1α, and SIRT3 have recently been identified as important components of an intracellular signaling pathway that regulates cellular energy output and mitophagy. AMPK plays a crucial role in regulating mitophagy mediated by PINK1 and Parkin [66]. When energy levels in cells drop, AMPK is activated and enhances PGC‐1α activity through direct phosphorylation [67]. PGC‐1α can directly promote the transcription of the SIRT3 gene by interacting with nuclear receptors [66], regulating mitochondrial biogenesis and energy metabolism [68]. Elevated SIRT3 expression and activity can deacetylate and activate several key proteins that regulate or facilitate mitophagy [69]. Research shows that the AMPK‐PGC‐1α‐SIRT3 pathway is important to honokiol's benefits against fluoride‐induced mitochondrial dysfunction, reducing central nervous system damage [22]. This mechanism is similar to AE's neuroprotective mechanism in ad observed in our study.

The commonly used traditional treatments for Alzheimer's disease (ad) include the following: cholinesterase inhibitors, which are used to increase acetylcholine (ACh) levels. These include drugs like donepezil, rivastigmine, and galantamine. NMDA receptor antagonists, which are used to modulate glutamate activity and reduce excitotoxicity, with memantine being the representative drug. Other adjunctive medications include nootropic drugs (such as piracetam) and antidepressants. Additionally, there are monoclonal antibody therapies (targeting the clearance of Aβ oligomers and protofibrils) and immunomodulatory therapies (activating microglial cells to enhance the clearance of Aβ plaques). In this article, we used the cholinesterase inhibitor donepezil as a positive control drug. Donepezil increases acetylcholine (ACh) levels. ACh improves autophagic flux by enhancing the expression of LC3II [70], and reduces ROS production through enhanced protective mitochondrial mitophagy, as shown in our results. Overall, most of donepezil's effects are mediated by the increase in ACh. In contrast, AE seems to target the upstream pathological cascades of Alzheimer's disease (ad). Specifically, our data, along with other studies [45], suggest that AE activates signaling pathways like AMPK/PGC‐1α/SIRT3, PI3K/AKT/mTOR, and NF‐κB. This improves mitochondrial bioenergetics and reduces oxidative stress and inflammation, key mechanisms in ad progression. The multitarget action contrasts with donepezil's single‐target approach, which may underlie the potential therapeutic effects in improving disease outcomes while minimizing adverse events.

Although this study presents important findings, there are still research gaps that need to be addressed. First, the results of this study indicate that the administration concentration of 50 mg/kg AE still shows significant effects on some indicators. This suggests that AE compounds may be sensitive to certain targets, and further exploration is needed in the future. Next, the results showed that the AE + siSIRT3 group still exhibited increased OPA1 levels and reduced Pink1/Parkin levels compared to the Model + siSIRT3 group. This suggests that, in the absence of SIRT3, AE still enhanced mitochondrial autophagy. This implies that AE may activate compensatory pathways rather than solely relying on the AMPK/PGC‐1α/SIRT3 pathway to regulate mitophagy. The potential mechanisms may include SIRT1‐mediated deacetylation (i.e., the synergistic effect of AMPK on the deacetylation of PGC‐1α [71] or Nrf2‐mediated antioxidant responses [72], which require further investigation). Additionally, Aloe‐emodin is a monomeric compound found in various TCM formulas, but it is seldom used in isolation following extraction. However, the interactions between different common TCM components remain unclear. Future research could explore the interactions of monomeric components in common TCM formulas to enhance clinical application.

In conclusion, we successfully demonstrated the neuroprotective effects of aloe‐emodin in vivo and in vitro ad models. Aloe‐emodin appears to provide neuroprotection in ad by activating mitophagy through the AMPK/PGC‐1α/SIRT3 pathway. Hence, our discoveries offer valuable insights for future research on the treatment of ad.

## Author Contributions

**Yulu Wang:** data acquisition, statistical analysis, and manuscript preparation. **Yunzhi Ge:** data analysis and manuscript preparation. **Siyu Hua:** literature search. **Chenrui Shen:** manuscript editing. **Biao Cai:** project administration and manuscript review. **Han Zhao:** study design and manuscript editing.

## Conflicts of Interest

The authors declare no conflicts of interest.

## Data Availability

The data will be available upon request.
